# Phase Velocity of Facial Blood Volume Oscillation at a Frequency of 0.1 Hz

**DOI:** 10.3389/fphys.2021.627354

**Published:** 2021-01-28

**Authors:** Kenichiro Yoshida, Izumi Nishidate

**Affiliations:** ^1^Kao Corporation, Skin Care Products Research, Tokyo, Japan; ^2^Graduate School of Bio-Applications and Systems Engineering, Tokyo University of Agriculture and Technology, Tokyo, Japan

**Keywords:** blood volume oscillation, phase velocity, cycle length, Mayer waves, vasomotion, digital camera imaging

## Abstract

Facial blood flow, which typically exhibits distinctive oscillation at a frequency of around 0.1 Hz, has been extensively studied. Although this oscillation may include important information about blood flow regulation, its origin remains unknown. The spatial phase distribution of the oscillation is thus desirable. Therefore, we visualized facial blood volume oscillation at a frequency of around 0.1 Hz using a digital camera imaging method with an improved approximation equation, which enabled precise analysis over a large area. We observed a slow spatial movement of the 0.1-Hz oscillation. The oscillation phase was not synchronized, but instead moved slowly. The phase velocity varies with person, measurement location, and time. An average phase velocity of 3.8 mm/s was obtained for several subjects. The results are consistent with previous studies; however, the conventional explanation that the blood flow at a certain point oscillates independently of adjacent areas should be corrected. If the primary origin of the movement is myogenic activity, the movement may ascend along a blood vessel toward the upstream. Otherwise, the oscillation and its propagation can be considered to be related to Mayer waves. By determining the mechanism, some questions regarding Mayer waves can be answered. The direction of the wave (upstream or downstream) provides important information.

## Introduction

Facial blood flow typically exhibits distinctive oscillation at a frequency of around 0.1 Hz (Lossius and Eriksen, 1995; Sasano et al., 1999; Perlitz et al., 2004; Bari et al., 2005; Nelson et al., 2006). Although this oscillation may reflect the conditions of circulation, its origin is unknown (Ticcinelli et al., 2015). Two possible origins have been postulated, namely blood pressure regulation during cardiovascular activity (Perlitz et al., 2004; Nelson et al., 2006; Cortez et al., 2019) and vasomotion with intrinsic oscillation during myogenic activity (Lossius and Eriksen, 1995; Söderström et al., 2003; Bari et al., 2005; Pradhan and Chakravarthy, 2011; Zamir et al., 2011; Ticcinelli et al., 2015, 2017). Oscillations measured at different points (even adjacent points) are often out of phase (Lossius and Eriksen, 1995), which does not support the oscillation originating from the heart. However, the oscillation is related to cardiovascular activity (Perlitz et al., 2004), which cannot be explained without involvement of the heart.

The derivation of a relationship between adjacent measurement points in terms of the blood flow oscillation phase will provide useful information. Regarding the cardiovascular origin, even if the oscillation phase between adjacent measurement points is very different, it does not mean that the oscillation between them is not linked (e.g., there may be a phase difference in the blood flow at different points). The spatial discontinuity of oscillation is inconsistent with the cardiovascular origin and may not support the myogenic origin. Assuming that blood flow oscillates within a certain vessel, it is reasonable to assume that the blood flow at the upstream and that at the downstream are linked. Considering that adjacent measurement points are connected *via* a blood vessel, the oscillation phases among adjacent points should be related; otherwise, blood supply from the upstream may be poor even when the blood vessel at a certain point is dilated. However, it is unknown to what extent the phenomenon is local and how a blood vessel is correlated with adjacent vessels.

According to a previous study (Lossius and Eriksen, 1995), oscillations between adjacent points are out of phase, which does not negate the possibility of a slow spatial movement of the phase along a blood vessel. However, such movement has not been reported. A heartbeat pulse wave with a frequency of about 1 Hz propagates at high velocity (about 10 m/s) (Yamashita et al., 2002; McEniery et al., 2005; Sugawara et al., 2005) through a blood vessel. If oscillations propagate at such velocity, the phase of a typical region of interest (ROI), such as a whole face, may seem to be almost synchronized. However, because blood vessels are viscoelastic (Learoyd and Taylor, 1966; Canić et al., 2006), blood velocity can vary largely with the frequency component of the blood pressure variation even if oscillations originate from the heart. For pulse waves with high- and low-frequency (0.1 Hz) variation, blood vessels may act as hard and soft walls, respectively. The latter may greatly affect wave velocity.

For these reasons, the spatial distribution of oscillation will provide important information. To visualize this distribution, we used digital camera imaging (DCI). We captured the spatial distribution of facial blood volume frame by frame and then concatenated the frames to obtain the spatiotemporal movement of blood volume. In DCI, the chromophore concentration is estimated from RGB values (Nishidate et al., 2013; Zaproudina et al., 2013; Yoshizawa et al., 2016; Okada et al., 2018). We modified a previously reported DCI method (Nishidate et al., 2013) in which the values are correlated with the actual concentration *via* Monte Carlo simulation. Laser Doppler flowmetry (LDF) is often used to measure oscillations (Lossius and Eriksen, 1995; Sasano et al., 1999; Perlitz et al., 2004; Bari et al., 2005; Nelson et al., 2006); however, it is unsuitable for spatiotemporal measurement. Nevertheless, because LDF measures blood flow whereas DCI measures blood volume, LDF was also used here to evaluate this difference.

In the DCI method, illuminance at each point must be estimated accurately to avoid systematic error. This is a difficult problem in practice for a large skin area (e.g., a whole face). Prior to conducting the study, we made the DCI method more suitable for large-area blood volume measurement. With our improvement, the effect of illuminance estimation error on the estimation of chromophore concentration is reduced given that time variation is of concern and the subject remains still during recording. We acquired videos of whole faces and derived moving images of oxyhemoglobin concentration. Then, we extracted moving images at a frequency of 0.1 Hz and used them to derive the phase velocity of waves. For comparison, LDF was used as a conventional method. Visible light spectroscopy (VLS) (Nishidate et al., 2004), which estimates blood volume at a point, was also used.

## Materials and Methods

### Laser Doppler Flowmetry

To measure the time course of blood flow at a certain point, a laser Doppler flow meter (ALF21D, Admedec Co. Ltd., Tokyo, Japan) was used with a type S probe (Admedec) and a probe holder. The time constant of the output was set to 0.1 s. The data acquisition software Chart (ADInstruments Inc., Springs, CO, US) was used. The sampling rate was set at 10 Hz. The unit of the measured blood flow was (mm/min/100 g).

### Visible Light Spectroscopy

To measure the time course of blood volume at a certain point, a previously reported measurement method (Nishidate et al., 2004) was used. A light source (HL-2000-HP, Ocean Insight, FL, USA) and a spectroscope (USB4000, Ocean Insight) were used. As a probe, a light guide (R400-7-UV-VIS, Ocean Insight), for which the diameter of each fiber was 400 nm and the center-to-center distance between the input and output fibers was 400 nm, was used with a probe holder. Spectra Suite (Ocean Insight) was used for data acquisition. The time course of the spectrum was recorded. The sampling rate was set to 10 Hz.

From the spectrum, the oxyhemoglobin concentration was derived at each moment as follows. First, the intensities in the wavelength range of 400–500 nm (in 10-nm intervals) were extracted from each measured spectrum and converted to reflectance under the assumption that the reflectance of a polytetrafluoroethylene standard white tile (WS-1; Ocean Insight) is one. Then, multiple regression analysis was performed with the reflectance spectrum as an objective variable and the absorption spectra of melanin, oxyhemoglobin, and deoxyhemoglobin as explanatory variables. From the derived multiple regression coefficients of these three chromophores and the constant term, the oxyhemoglobin concentration was estimated using a third-order equation that was derived in advance. We used the oxyhemoglobin concentration in the analysis because we focused on arterial blood. The estimation equation was derived to fit the results of Monte Carlo simulation. The blood was assumed to have a hematocrit of 44%, and an oxyhemoglobin concentration of 150 g/l in the dermis was defined as 100%.

### Digital Camera Imaging

**Figure 1:**
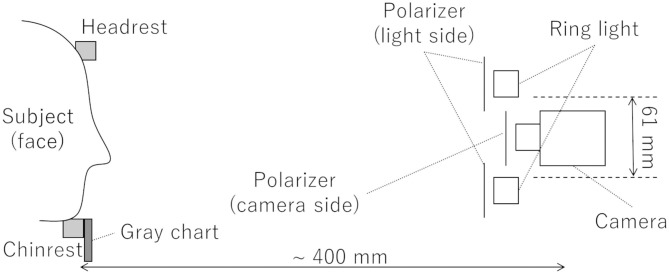
Schematic diagram of video system used for DCI.

To measure the time course of blood volume as a sequence of images, a previously reported measurement method (Nishidate et al., 2013) was used. A schematic diagram of the video system used for DCI is shown in Figure 1. A light source (LA-HDF158AS, Hayashi-repic, Tokyo, Japan) was used with a light guide (LGB1-8L1000-R61, Hayashi-repic; ring type, inner diameter: 61 mm). A video camera (DFK23UM021, The Imaging Source, Bremen, Germany) was used with a 6-mm lens (FL-HC0612A-VG, Ricoh, Tokyo, Japan). Each component was firmly anchored to an optical rail. Polarization plates were set in front of the light guide and the camera in such a way that the polarization directions were perpendicular to each other to exclude surface reflection from the videos. The camera was set so that the top of the lens was placed at the center of the light guide ring. The distance between the camera and the chinrest was about 400 mm. The frame rate was set to 23.77 frames/s (FPS). Videos were recorded as uncompressed Audio Video Interleave (AVI) files. A gray chart placed under the chinrest was used for exposure correction.

From a recorded video, illuminance correction was executed frame by frame with the gray card image in each frame. Then, after the application of a 3 × 3 average filter to each frame, the frame rate was halved to reduce noise and data size; a new frame was made by averaging two successive frames pixel by pixel and channel by channel (RGB). The new frame rate (11.89 FPS) had sufficient temporal resolution for observing 0.1-Hz blood volume oscillation. The derived images were used as input for the chromophore concentration estimation.

From the video-derived images, the oxyhemoglobin concentration was estimated frame by frame and pixel by pixel, with RGB values converted to values in the XYZ color system. The concentration was derived from these XYZ values. We used oxyhemoglobin concentration, as done in VLS, because we focused on arterial blood. A previously reported equation (Nishidate et al., 2013), briefly described in the appendix, was used as a base. In the original method, illuminance cannot be accurately estimated pixel by pixel when the ROI is large or uneven or its shape is subject-dependent. We improved the method to eliminate this problem given that the subject remains still during video recording and only temporal variation is of concern (not the absolute value). This improved method is also described in the appendix. In practice, M_1_ “in the appendix (Supplementary Material)” was derived to make the XYZ values of color charts (ColorChecker; X-rite, Grand Rapids, MI, USA) match the ones described in the specifications of the color charts provided by the supplier. M_2_' “in the appendix (Supplementary Material)” was derived utilizing Monte Carlo simulation and had values of 11.13525, −10.0444, −0.33082, and −2.57943. The difference between the values estimated using the original equation and the improved equation was small enough under the condition that illuminance is well estimated, which was confirmed in a preliminary test. The unit of oxyhemoglobin concentration was (ml/100 ml).

For quantification, several ROIs (left cheek, right cheek, and forehead) were set visually for each series of frames. The ROIs were fixed over a series of frames. All ROIs were the same size (20 × 20 pixels; actual size of 7.3 mm × 7.3 mm on a subject). The average oxyhemoglobin concentration in each ROI was calculated frame by frame.

### Analysis

#### Platform and Flow of Data Processing

**Figure 2:**
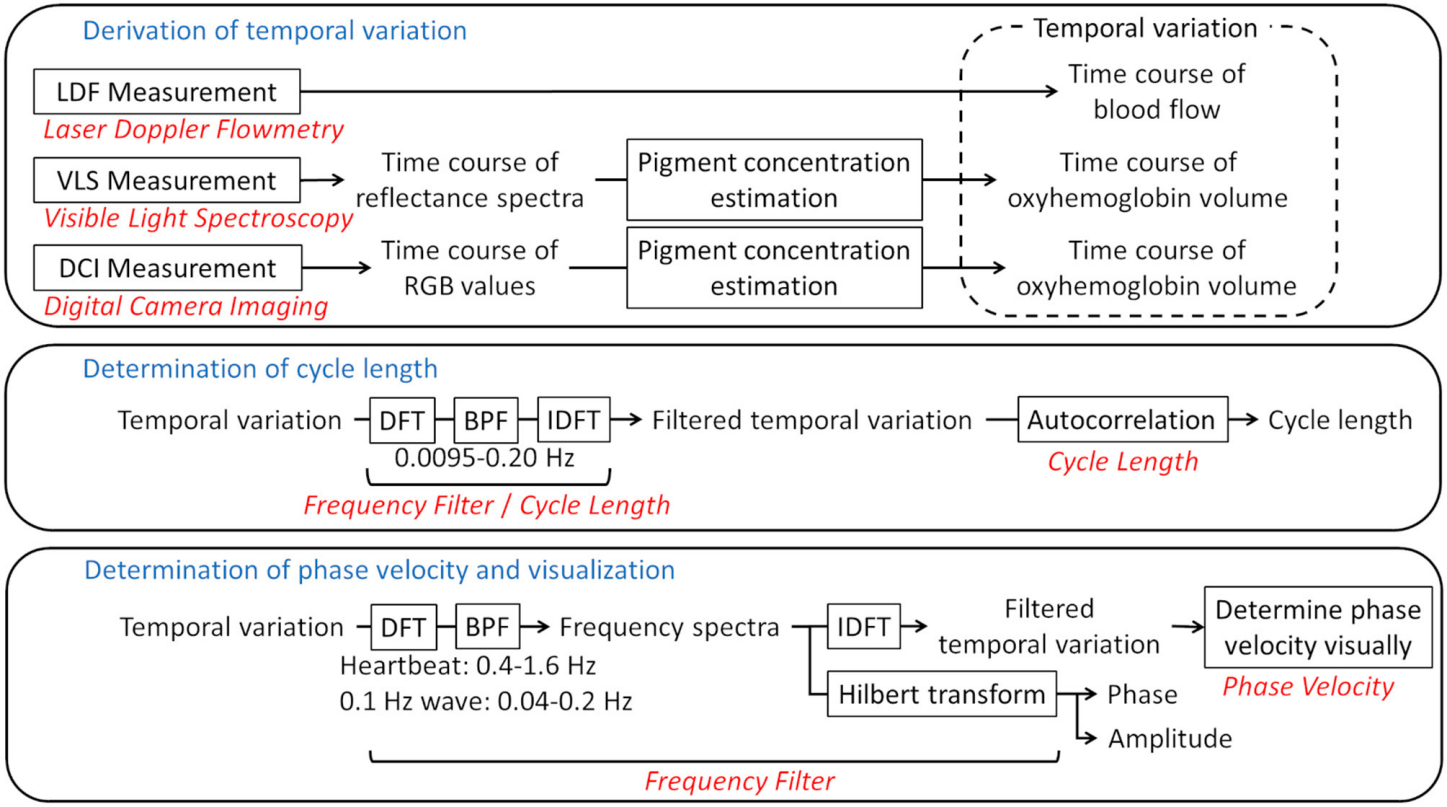
Flow chart of the analysis. Framed rectangles represent data processing steps. The relevant sections in the text are written in red. The frequency ranges of the bandpass filters are also provided. LDF, laser Doppler flowmetry; VLS, visible light spectroscopy; DCI, digital camera imaging; DFT, discrete Fourier transform; BPF, bandpass filter; IDFT, inverse discrete Fourier transform.

MATLAB (MathWorks, Natick, MA, US) with the Image Processing Toolbox (MathWorks) was used as the platform for analysis. Figure 2 shows a flow chart of the data processing.

#### Frequency Filter

To acquire the time variation for a certain frequency range, the discrete Fourier transform (DFT) was applied to a time course with the hamming window as a window function. Bandpass filters were then applied, followed by the inverse DFT. To make the phase of oscillations comprehensible, the Hilbert transform was applied to the spectrum and the phase and amplitude for the bands of interest were derived. The bands were set to 0.4–1.6 Hz for the heartbeat and 0.04–0.2 Hz for the 0.1-Hz wave. The range of the 0.1-Hz wave was defined so that the entire peak of the 0.1-Hz wave was included in the power spectrum.

#### Cycle Length

To estimate a cycle length, autocorrelation was used. The autocorrelation of a certain time course *I*(*t*) was defined as:

(1) $$\begin{array}{l} A\left(\tau \right)=\frac{\sum_{t}\left(I\left(t\right)\times I\left(t-\tau \right)\right)}{\sum_{t}{I\left(t\right)}^{2}}. \end{array}$$

**Figure 3:**
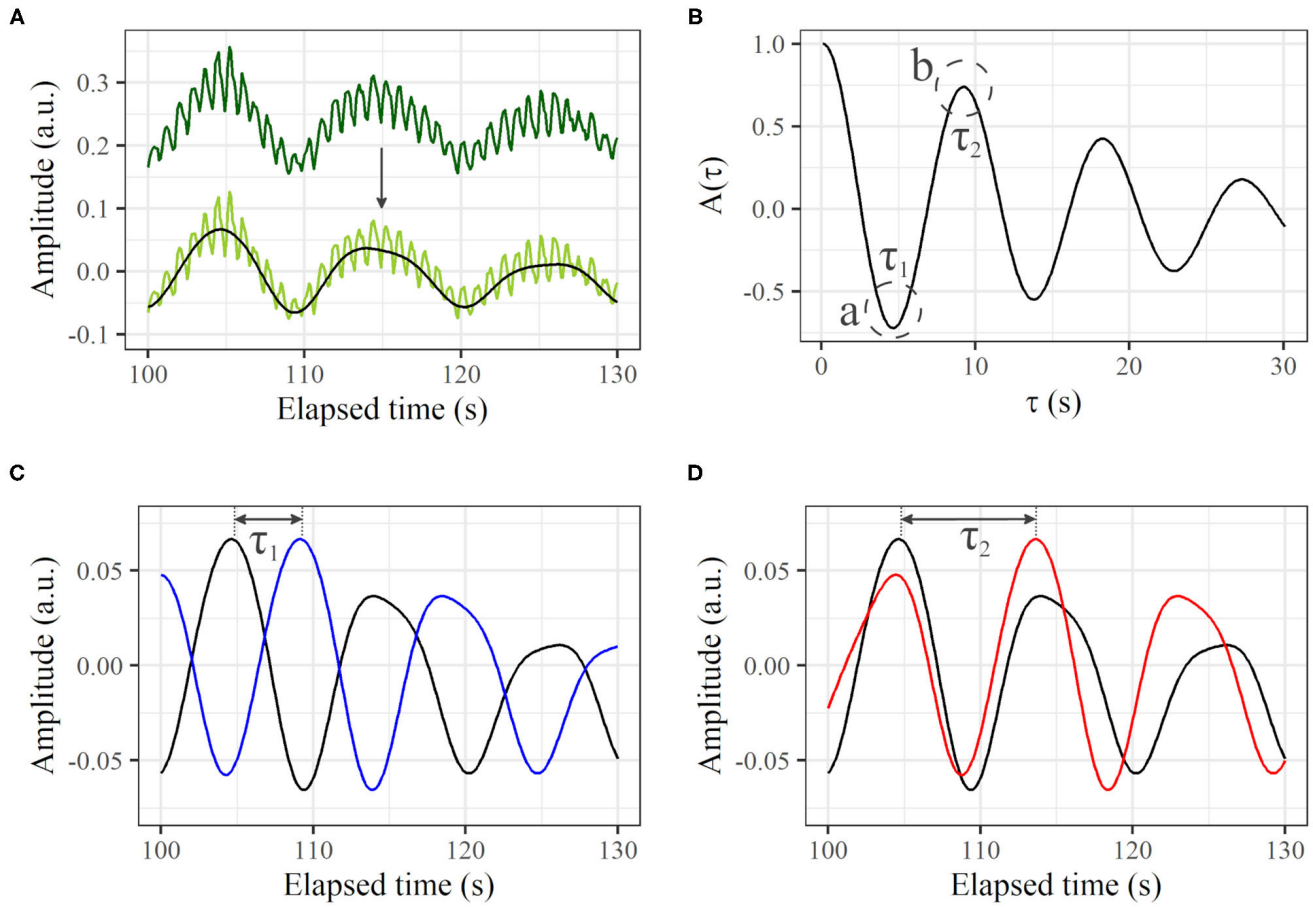
Autocorrelation based on a time course of LDF. **(A)** Thirty seconds of profile from original time course (dark green line), profile with direct-current component removed (light green line), and extracted signal in frequency range of 0.0095–0.20 Hz (black line). **(B)** Calculated A(τ). The first local minimum and maximum of A(τ) are τ_1_ and τ_2_, respectively. **(C)** Original time course [black line; same as black line in **(A)**] and τ_1_-shifted profile (blue). **(D)** Original time course [black line; same as black line in **(A)**] and τ_2_-shifted profile (red).

From a profile of the time course (Figure 3A, dark green line), the direct-current component was removed (Figure 3A, light green line). The heartbeat component was reduced by the 0.0095–0.20 Hz bandpass filter (Figure 3A, black line). If a certain variance *I*(*t*) oscillates regularly to some extent over time like this, *A*(τ) decreases with τ from τ = 0, and reaches a local minimum negative value when τ is at the half-cycle length (τ_1_ in Figure 3B-a). Conversely, from τ_1_, *A*(τ) increases with τ, and reaches a local maximum positive value when τ is at the one-cycle length (τ_2_ in Figure 3B-b). For this example, τ_1_- and τ_2_-shifted time courses overlaid on the original time course are shown in Figures 3C,D, in which we can visually recognize that τ_1_ and τ_2_ represent the half- and one-cycle lengths, respectively. Therefore, we define τ_2_ as the cycle length if *A*(τ_1_) < 0 and *A*(τ_2_)>0, where τ_1_ is τ at the first local minimum from τ = 0 and τ_2_ is τ at the first local maximum. If there are no τ_1_ and τ_2_ that satisfy such conditions, the cycle length is considered to be indeterminate. *A*(τ) is calculated from τ = 0 to τ = 30 s, and the summation is conducted over the measurement time. Although the peak of the power spectrum in the frequency domain is often used for the cycle length, we used the autocorrelation technique because the profile of the autocorrelation is smooth compared to the profile of the power spectrum, making the derivation of cycle length easy.

#### Phase Velocity

With the phase expression in degrees (0, 360°), the distance on an image between the phases 90 and 180° (or 90 and 270° depending on visibility) along the wave direction was measured visually. Then, the value multiplied by four (two) was regarded as the spatial distance of one cycle. The temporal cycle length was also derived. The phase velocity was derived by dividing the spatial distance of one cycle by the temporal cycle length. The forehead was used to derive the phase velocity because it is relatively flat and faces the front.

## Experiments

This study was reviewed and approved by the Human Research Ethics Committee of Kao Corporation. The participants provided written informed consent to participate in this study. Healthy Japanese females (*n* = 40) in their 30 s (*n* = 10), 40 s (*n* = 20), and 50 s (*n* = 10) participated. For each participant, after acclimatization for more than 10 min at 24°C and 50% relative humidity at rest, the following measurements were conducted. First, with the participant seated on a reclining chair, probes were attached to their respective sites, and VLS spectra (at right cheek) and LDF signals (at left cheek) were recorded for 15 min. The slope of the backrest was about 60° with respect to the horizontal. Next, in a dark room, the participant was seated on a chair and placed her head on the chinrest. Then, with the participant keeping still, a video of the face was recorded for 5 min using DCI.

## Results

**Figure 4:**
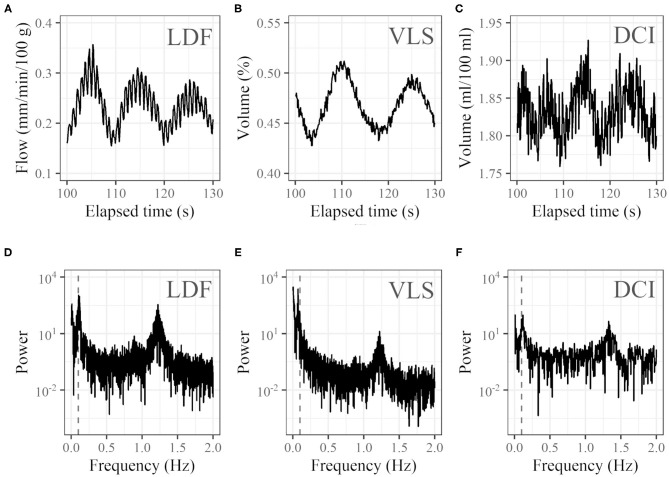
Examples of profiles. Thirty seconds of time courses of **(A)** LDF (blood flow at left cheek), **(B)** VLS (oxyhemoglobin concentration at right cheek), and **(C)** DCI (oxyhemoglobin concentration at right cheek) and **(D–F)** corresponding power spectra in the frequency domain (vertical dashed gray lines represent 0.1 Hz). For the time course of DCI, the average of the 20 × 20 pixel (7.3 mm × 7.3 mm) ROI was used.

Representative 30-s examples clipped from the temporal variation of LDF, VLS, and DCI are shown in Figures 4A–C. Respective power spectra from the full-length temporal variation in the frequency domain are shown in Figures 4D–F. For many subjects, noticeable oscillation at around 0.1 Hz was observed in addition to a peak caused by the heartbeat at around 1 Hz. However, for some subjects, no such oscillation was observed.

**Figure 5:**
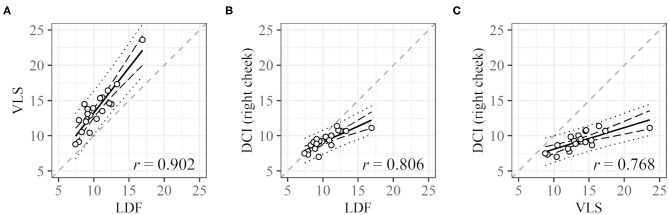
Correlation between cycle lengths [s] obtained with different measurement methods. **(A)** LDF vs. VLS, **(B)** LDF vs. DCI, and **(C)** VLS vs. DCI. Dashed black lines and dotted black lines represent the confidence interval and prediction interval, respectively. Dashed gray lines represent the lines *X* = *Y*. Only data for subjects with all three values were used.

For one subject, LDF and VLS data could not be obtained because of measurement failure. Cycle lengths could be derived for 30 (LDF), 34 (VLS), and 25 (DCI) of the remaining 39 participants. For 20 subjects, three cycle lengths based on LDF, VLS, and DCI could be derived. The plots of the cycle lengths of these 20 subjects obtained using different measurement methods are shown in Figure 5. Note that LDF and VLS were measured simultaneously at the left and right cheeks, and DCI was measured separately. The values obtained from VLS are slightly higher than those obtained from LDF. The slopes are near one and have a high correlation. The values obtained from DCI are lower than those obtained from LDF and VLS, and their slope is below one, but the correlations are still high.

**Figure 6:**
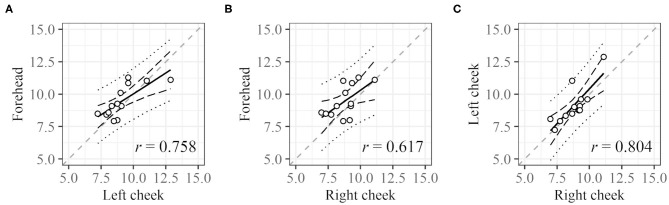
Correlation between cycle lengths of different regions. **(A)** Left cheek vs. forehead, **(B)** right cheek vs. forehead, and **(C)** right cheek vs. left cheek. Dashed black lines and dotted black lines represent the confidence interval and prediction interval, respectively. Dashed gray lines represent the lines *X* = *Y*. Only data for subjects with all three values were used.

For DCI, the cycle length at each ROI was compared (Figure 6). Cycle lengths could be derived for 25 (right cheek), 25 (left cheek), and 21 (forehead) subjects. For 13 subjects, cycle lengths were derived for all three sites (for 31 subjects, cycle lengths could be measured for at least one ROI). The plots of the cycle lengths of these 13 subjects obtained at different sites are shown in Figure 6. The cycle lengths for different ROIs are similar.

**Figure 7:**
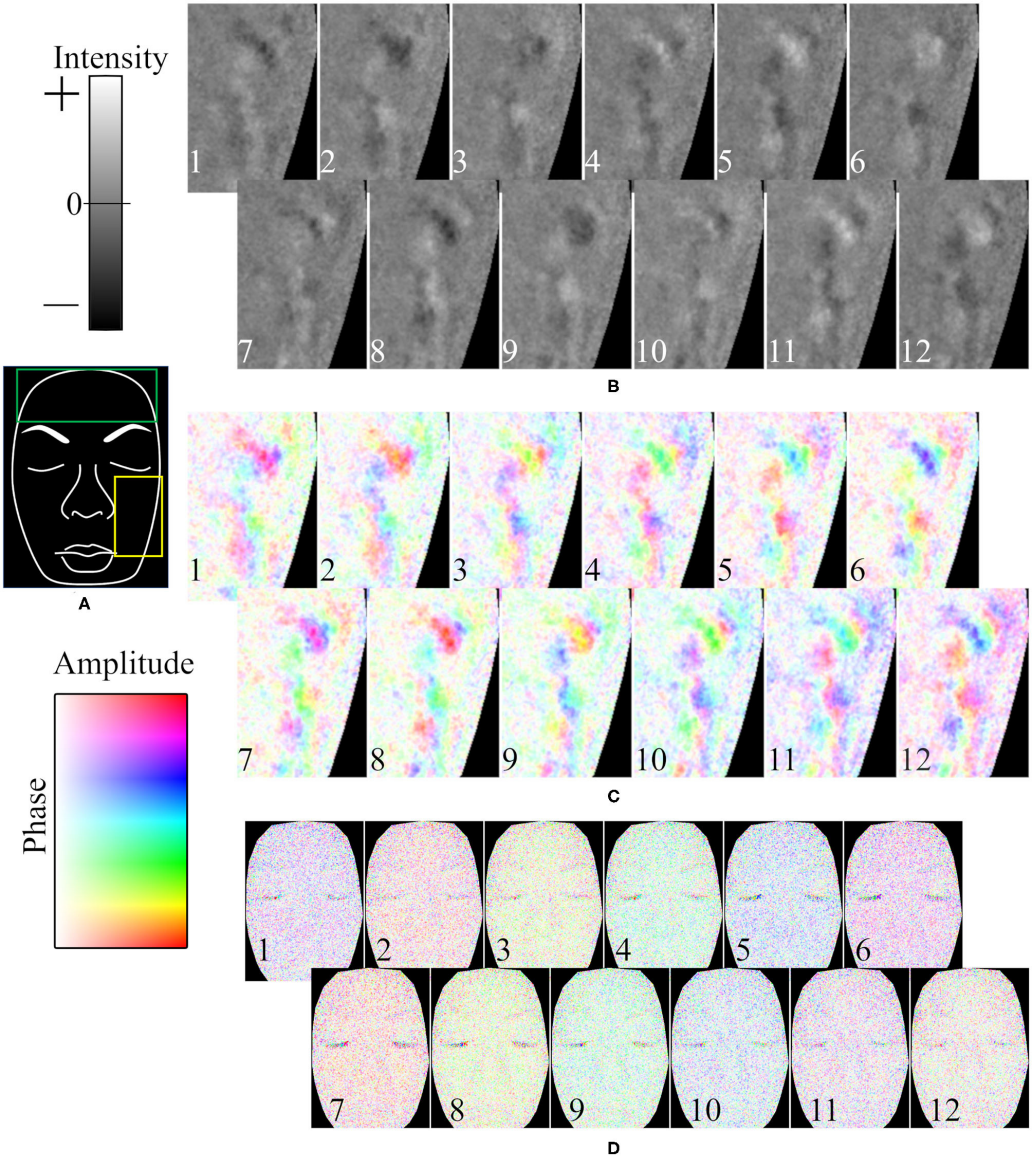
Representative example of time course of oxyhemoglobin image. **(A)** Hand-drawn copy of an original image. Time course of oxyhemoglobin image in **(B)** 0.04–0.2 Hz frequency range for intensity, **(C)** 0.04–0.2 Hz frequency range for phase and amplitude, and **(D)** 0.4–1.6 Hz frequency range for phase and amplitude. The yellow rectangle in **(A)** is the ROI in **(B,C)**. The time differences between neighboring frames (duration from the 1 to 12th frames) is **(B,C)** 1.26 s (13.86 s), and **(D)** 0.168 s (1.85 s). The numbers in **(B**–**D**) represent the time course.

A representative example of the temporal variation of the spatial phase distribution is shown in Figure 7. Figure 7A is a hand-drawn copy of a frame from a video. The time course of the intensity in the 0.1-Hz frequency range of oxyhemoglobin around the left cheek is shown in Figure 7B. As shown, the rise and fall did not simultaneously occur across the whole face; instead, the wave moved slowly across the surface. In this example, the patterns of the first and eighth images (a temporal difference of about 8.8 s), second and ninth images, and so on, are similar. Figure 7C shows the same data given in Figure 7B but subjected to the Hilbert transform. It should be noted that the oscillation was not continuously observed during the measurement; instead, it emerged at one moment and then vanished, and such oscillation did not occur at all for some subjects.

The time course of the phase in the 1-Hz frequency range of oxyhemoglobin was synchronized across the whole face, as shown in Figure 7D. In the example, the patterns (colors) of the first and sixth images (a temporal difference of about 0.84 s), second and seventh images, and so on, are similar.

**Figure 8:**
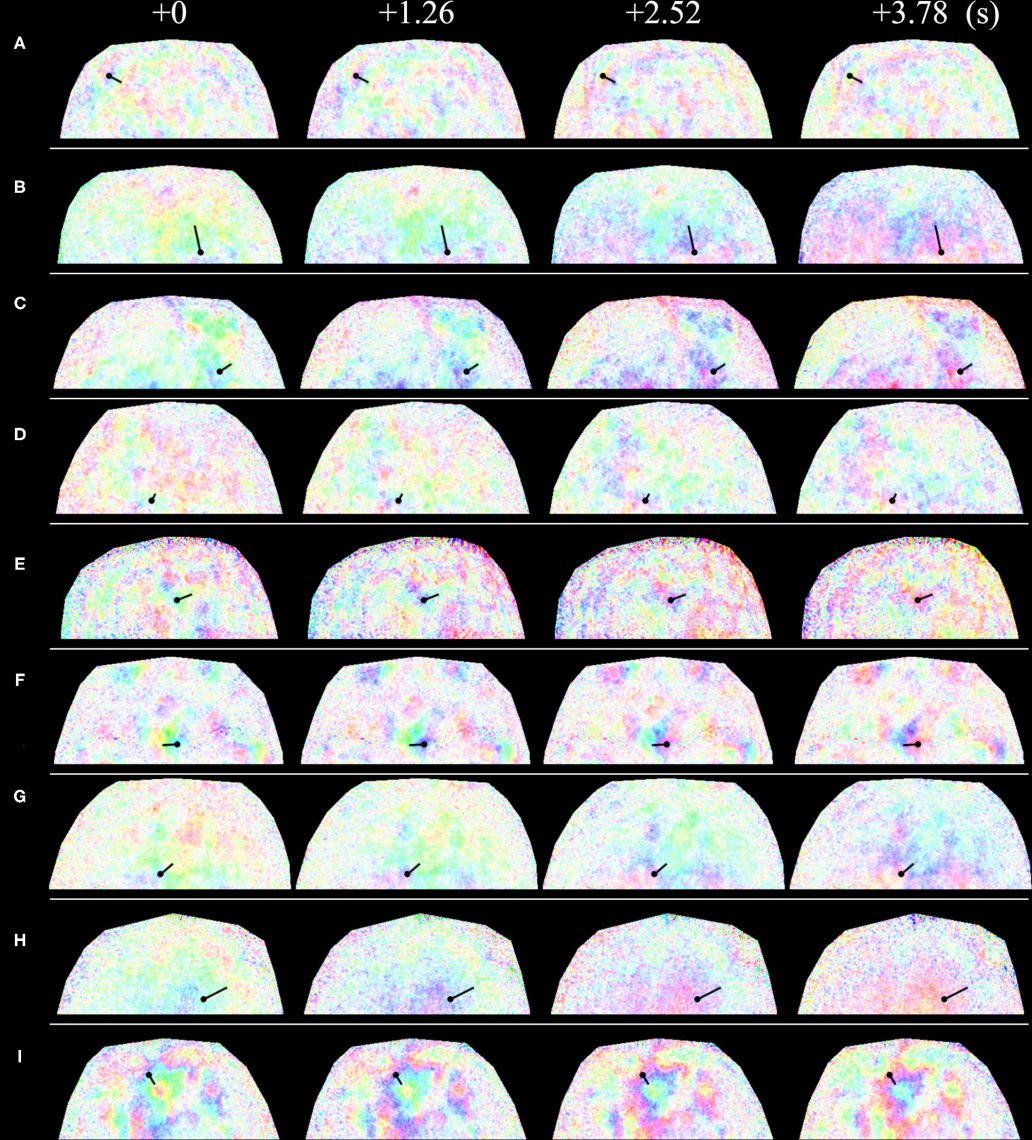
Time course of spatial phase distribution of 0.1-Hz component of blood volume variation at the forehead. Straight solid lines represent the measurement location, where waves moved from the side with the black circle to the side without the black circle. The phase difference from one side to the other side of the straight lines is **(A)** 180° and **(B–I)** 90°. The relationship between color and phase and amplitude is the same as that in Figure 7. **(A–I)** show images for different subjects.

The concrete values of phase velocity varied with subject, location, and time. The derived phase velocities for several examples are shown in Figure 8 and Table 1. The spatial intervals of the half phase or quarter phase at a certain frame on the forehead are shown in Figure 8. The derived phase velocities in Table 1 were calculated.

**Table 1:** Phase velocities (PVs) calculated at the respective space-time points in Figure 8.

**Site**	**A**	**B**	**C**	**D**	**E**	**F**	**G**	**H**	**I**	**Average**
PV (mm/s)	1.79	6.38	6.31	2.70	4.81	4.24	3.87	2.42	1.29	3.76

## Discussion

In this study, an intense 0.1-Hz oscillation of oxyhemoglobin concentration often appeared on the face. It often had an extremely slow phase velocity compared to the pulse velocity (Figure 7). In this case, adjacent points had different phases, which is consistent with a previous study (Lossius and Eriksen, 1995); however, the phases were spatially continuous, which has not been previously reported. The oscillation was not always stable and sometimes disappeared or emerged, which is consistent with previous studies (Lossius and Eriksen, 1995; Bari et al., 2005).

In terms of measurement parameters, in the applied DCI, the derived value is associated with blood volume, whereas in LDF, which was frequently used in previous studies, the derived value is blood flow. Nevertheless, we think that the results obtained from DCI are basically comparable. First, the cycle lengths of the 0.1-Hz oscillation in DCI had a strong relationship with those obtained in LDF (Figure 5). The cycle lengths in DCI are smaller than those in LDF probably because of posture differences. The subjects laid on a reclining chair comfortably in LDF whereas they sat on a chair without a backrest and kept their chin on a chinrest in DCI, which introduced some strain. Such strain resulted in a shorter cycle length through vascular contraction. Second, the values of cycle length from VLS and LDF, which were obtained simultaneously, were similar (Figure 5). The measured value in VLS is blood volume, as in DCI. This indicates that variations in blood flow and blood volume are comparable for evaluating the oscillation considered in this study. In addition, the pulse wave was detected in DCI, which supports the validity of the method. According to previous studies on pulse wave velocity (PWV) (Yamashita et al., 2002; McEniery et al., 2005), a pulse wave is fast enough (~10 m/s) to be detected as the same phase across a whole face by our instrument (23.77 FPS), which is consistent with our results (Figure 7).

The observed phases of the oscillation among various measurement points did not match; however, they were harmonized and had a constant difference. Although this result may not eliminate the vasomotion origin, it cannot be stated that the oscillation is unrelated to cardiovascular blood pressure regulation because it was harmonized globally if the oscillation was moving downstream. It is unlikely that the oscillation is unrelated to cardiovascular activity even if the main origin is myogenic activity because the 0.1-Hz oscillation exhibited global movement and its frequency range was coincident with that of cardiovascular oscillation or Mayer waves (Julien, 2006, 2019; Molkov, 2014).

If the oscillation originates from the heart, the phase velocity is extremely slow compared with that of a heartbeat. This may be explained by the effect of vasomotion (Malpas, 2002); however, it can also be explained by the viscoelasticity of blood vessels. Consider the Moens–Korteweg equation (Gosling and Budge, 2003; Boutouyrie et al., 2009), which is commonly used to describe pulse propagation velocity (there is no equation for Mayer wave propagation). The equation is $PWV^{2}=\frac{Eh}{2r\rho }$, where *E, r*, and *h* are respectively the elastic modulus, radius, and thickness of a blood vessel and ρ is the blood density. From the equation, we can see that if *E* is small, PWV becomes small. Because blood vessels are viscoelastic (Learoyd and Taylor, 1966; Canić et al., 2006), they act as a soft material for slow pressure variation due to stress relaxation. We hypothesize that the instability of oscillation originates from the variation of the physical properties of blood vessels (e.g., elastic modulus and radius). The properties of blood vessels on the face seem more favorable for stable oscillation than, for example, those of vessels on the arm, where such oscillation is seldom observed, but they are insufficient for making the oscillation completely stable.

Although many questions remain about the origin and mechanism of Mayer waves (Julien, 2006, 2019; Molkov, 2014), if the observed blood volume oscillation reflects the movement of Mayer waves, it may resolve some of the uncertainties associated with these waves. The main question for Mayer waves is the 0.1-Hz cycle length, which is too long with respect to the time scale of nervous system activity. Many attempts have been made to determine the feedback loop and explain the 0.1-Hz oscillation; however, the proposed feedback loops tend to be unstable or stable in a limited range of parameters (deBoer et al., 1987; Malpas, 2002). In many studies, systolic arterial pressure (or mean arterial pressure), which reflects the shape of each pulse, was equated to blood pressure as an input to baroreceptors, where its propagation along with pulse was implicitly assumed. If baroreceptors respond to the swell caused by the average blood pressure, and if the swell moves slowly from the heart to baroreceptors, as observed on faces, the oscillation may arrive at baroreceptors much later than the wave of systolic arterial pressure. By embedding the travel time of waves from the heart to baroreceptors in the feedback loop of the oscillation, the long cycle length may be determined.

Another major question for Mayer waves is the difference in cycle length between species (Malpas, 2002). The cycle length of Mayer waves is about 0.7 Hz for mice, 0.1 Hz for humans and cats, and 0.3 Hz for rabbits according to previous studies (Julien, 2006, 2019). Because we considered only the transfer of nerve signals as an element of the feedback loop, it is difficult to explain this difference. Because the time lag between the input and output is very short and the speed of propagation is very fast, even including a scale factor for body size cannot explain the difference. However, it may be explained by introducing the duration of the 0.1-Hz oscillation propagating from the heart to baroreceptors, which is slow and affected by the distance between the heart and baroreceptors.

It is possible that the oscillation originates only from myogenic activity and not from Mayer waves. During myogenic activity, vascular smooth muscles contract when vascular pressure increases (Söderström et al., 2003). Therefore, if a certain part of a blood vessel contracts, the vascular pressure at the adjacent upstream may increase and the blood vessel there may contract, which may explain the observed phenomenon. According to this explanation, the waves may go upstream. The contribution of myogenic activity to the response to blood demand is known (Zamir et al., 2011), but it has not been observed *in vivo* as a global movement. If the movement is toward the upstream, it would support this explanation. If the movement is toward the downstream, it is likely to be caused by the heart. Therefore, the direction of the wave movement (upstream or downstream) will provide important information. Further study is thus required.

It is worth confirming that the phenomenon we discussed is specific to human faces. In general, the 0.1-Hz component in time-frequency analysis of blood flow in the peripheral vasculature is recognized as vasomotion with a myogenic activity origin, which is supported by much evidence. For example, a study of blood flow on surfaces of free microvascular flaps found that frequency intensities of blood flow rhythm were significantly lower for signals measured on the free flap and the peaks almost disappeared; however, only the 0.1-Hz component remained (Söderström et al., 2003). In another study, capillary blood flow in hamster skeletal muscle microcirculation was observed by microscopy, and the frequency of blood flow oscillation differed according to the vessel branching order (Lapi et al., 2019). The phenomena just described cannot be explained with a cardiovascular oscillation origin. However, the appearance of the 0.1-Hz oscillation on a face is substantially different from most of those that appear on other sites and is strong enough that it can be easily recognized even without frequency analysis (Figure 4). Therefore, it is reasonable to expect that there exists some unique mechanism behind this oscillation. We used a 0.0095–0.20 Hz bandpass filter for the cycle length derivation. The range of this filter includes all frequency ranges of myogenic activity (typically 0.052–0.145 Hz), neurogenic activity (0.021–0.052 Hz), and endothelial activity (0.0095–0.021 Hz), which are commonly used in frequency analysis of vasomotion (Söderström et al., 2003; Rossi et al., 2012; Tankanag et al., 2014). The cycle length can be derived for many participants, which is an indication of how strong the 0.1-Hz oscillation is.

A comparison of the time course of DCI (Figure 4C) to that of VLS (Figure 4B) indicates that the noise of DCI is larger, which can be explained as follows. First, in DCI, the distance between the incident point and the measurement point in terms of the probe light propagation is not controlled, which is important for extracting information at a certain depth. It is controlled in VLS based on the form of the probe. Another reason is the limitation of color depth, which is 8 bits (256 levels) for each RGB channel in DCI. In VLS, the spectrum is acquired at 12 bits per channel. Additionally, in DCI, the intensity of light and the gain were adjusted for the whole image during acquisition, which makes effective utilization of the dynamic range difficult and decreases intensity resolution. The color-depth limitation can be reduced by using a higher depth (e.g., 16 bits), which would allow more precise measurement.

The focus of the present study was on observing the phenomenon, so no attempts were made to correlate the oscillation with any physical or physiological conditions or to evaluate changes after any interventions. These are issues for future consideration. In addition, the participants were limited to Japanese females [Asian; Types III and IV of the Fitzpatrick scale (Fitzpatrick, 1988)], and studies of the phenomenon in other ethnicities and males are needed. A concern regarding the range of application of DCI, such as for dark skin (e.g., Type VI of the Fitzpatrick scale), is that the estimation error could exceed the application range if there is too little reflectance from the skin. This is a common problem among methods of analysis that utilize the shape of the visible light reflectance spectrum. Assessment of the application range in terms of skin color is also a future area of study.

## Conclusion

In this paper, we visualized facial blood volume oscillation at a frequency of around 0.1 Hz. Different from the phase of heartbeats, the phase of the oscillation is not synchronized but instead moves slowly. In point-by-point measurements, adjacent points had different phases, which is consistent with a previous study; however, the phases were spatially continuous, which has not been previously reported. The results did not eliminate vasomotion as the possible origin of the oscillation. If this oscillation is truly independent of Mayer waves, we should accept the idea that the spatial movement of oscillation is the propagation of vasomotion across a large area. We think that it is more reasonable to consider that the oscillation and its propagation are related to Mayer waves. Determining the mechanism may clarify some uncertainties regarding Mayer waves.

The present study uncovered the spatial movement of facial blood volume oscillation that is difficult to explain as purely vasomotion with current knowledge. Our conclusion needs to be verified, but offers some valuable insight into the hemodynamics of the face and the importance of this characteristic that has not been well accounted for in the literature.

## Data Availability Statement

The raw data supporting the conclusions of this article will be made available by the authors, without undue reservation.

## Ethics Statement

The studies involving human participants were reviewed and approved by Human Research Ethics Committee of Kao Corporation. The patients/participants provided their written informed consent to participate in this study.

## Author Contributions

KY and IN contributed to the methodology. KY contributed to the conception, analysis, and writing. KY and IN contributed to manuscript revision and approved the submitted version. All authors contributed to the article and approved the submitted version.

## Conflict of Interest

KY is an employee of Kao Corporation. The remaining author declares that the research was conducted in the absence of any commercial or financial relationships that could be construed as a potential conflict of interest.
