# Supplementary material for: Phase Velocity of Facial Blood Volume Oscillation at a Frequency of 0.1 Hz
Source: Front Physiol. 2021 Jan 28;12:627354. doi: 10.3389/fphys.2021.627354 (PMC7876320; doi:10.3389/fphys.2021.627354)
Supplement: Supplementary file 1 [file Data_Sheet_1.pdf]

[Appendix] Phase velocity of facial blood volume oscillation at a frequency of 0.1 Hz

Kenichiro Yoshida[1*], Izumi Nishidate[2]

[1] Kao Corporation, Skin Care Products Research, Tokyo, Japan

[2] Tokyo University of Agriculture & Technology, Graduate School of Bio-applications & Systems Engineering, Tokyo, Japan

*** Correspondence:**
Kenichiro Yoshida, Kao Corporation, Skin Care Products Research, 2-1-3 Bunka, Sumida-ku, Tokyo 131-8501, Japan
yoshida.kenichiro@kao.com

A Appendix

In general, if the number of unknown variables is larger than the number of measured variables, the unknown variables cannot be determined uniquely. In our approach, which determines the chromophore concentrations of melanin, oxyhemoglobin, and deoxyhemoglobin from RGB values at each pixel, the numbers of unknown and measured variables are both three, and thus the problem is solvable. However, if the illuminance distribution is unknown, the number of unknown variables becomes four, and then the problem becomes unsolvable. For a large area of human skin (e.g., a whole face), whose shape is uneven and varies between individuals, it is difficult to estimate illuminance precisely. For such cases, the estimation error of illuminance will directly affect the estimation error of chromophore concentrations. However, we found that the estimation error of illuminance can be removed by changing the type of approximation formula for chromophore concentrations when our concern is the variance of each point on the skin. Using the improved formula, the variations of chromophores are not affected by the estimation error of illuminance given that the subject does not move during recording.

A.1 Original formulas

First, we express the pixel values at a certain point in an image of skin as RGB values under the assumption that they are proportional to the light intensity and that white balance correction has been applied. The pixel values can be transformed to the XYZ color system (ISO 11664-1) using matrix M_1, which is determined according to the wavelength sensitivity of a camera.

$$\begin{pmatrix} X \\ Y \\ Z \end{pmatrix} = M_1 \cdot \begin{pmatrix} \alpha \cdot R \\ \alpha \cdot G \\ \alpha \cdot B \end{pmatrix}. \tag{A.1}$$

Here, α represents the coefficient for the conversion to reflectance. It must be determined pixel by pixel if illuminance is uneven.

Supposing a specific optical model of skin, we set up a problem to determine the correlation between the chromophore concentration and the color of reflected light (i.e., the values of XYZ). Here, we

chose melanin, oxyhemoglobin, and deoxyhemoglobin as the chromophores. Their concentrations are respectively represented as C_m, C_{oh}, and C_{dh}. By varying C_m, C_{oh}, and C_{dh} under the optical model, reflectance spectra can be derived from Monte Carlo simulation. Then, XYZ can be derived using a color-matching function. With the sets $(C_m \quad C_{oh} \quad C_{dh})$ and $(X \quad Y \quad Z)$, a linear approximation formula can be derived for each chromophore from XYZ. More specifically, the formulas were derived with multiple linear regression analyses using C_m, C_{oh}, and C_{dh} as objective variables and X, Y, and Z as explanatory variables. We explain the process with the formula for C_{oh}, which was used in this paper. The formula can be expressed as:

$$C_{oh} = M_2 \cdot \begin{pmatrix} X \\ Y \\ Z \\ 1 \end{pmatrix}, \tag{A.2}$$

where M_2 is a row vector.

The value of α is affected by the spatial distribution of illumination and the shape of a subject, which varies from person to person. The difference among individuals becomes crucial if the ROI is large. Here, we assume that $\alpha' = k \cdot \alpha$ is used as the coefficient instead of the actual value (α). The derived concentration C'_{oh} can then be expressed as:

$$C'_{oh} = M_2 \cdot \begin{pmatrix} k \cdot X \\ k \cdot Y \\ k \cdot Z \\ 1 \end{pmatrix} = k \cdot C_{oh} + (1 - k) \cdot M_2 \cdot \begin{pmatrix} 0 \\ 0 \\ 0 \\ 1 \end{pmatrix}. \tag{A.3}$$

If the subject remains still during video recording, k at each pixel is constant over time; therefore, the second term is constant, which means that the temporal variation is in the first term and the estimation error appears as the product of k and C_{oh}. In other words, the amplitude of temporal variation is strongly influenced by subject shape.

A.2 Improved formula

Instead of Eq. (A.2), the following formula was used as the approximation formula:

$$C_{oh} = M_2' \cdot \begin{pmatrix} \log(X) \\ \log(Y) \\ \log(Z) \\ 1 \end{pmatrix}. \tag{A.4}$$

By doing so, the absolute values are still affected by the estimation error of illuminance, but the amplitude of temporal variation is not. This can be explained as follows.

If we again assume that $\alpha' = k \cdot \alpha$ is used as the coefficient instead of α, using the general relationship $\log(A \cdot B) = \log A + \log B$, the derived concentration C'_{oh} can be expressed as:

$$C'_{oh} = M_2{}' \cdot \begin{pmatrix} \log(k \cdot X) \\ \log(k \cdot Y) \\ \log(k \cdot Z) \\ 1 \end{pmatrix} = C_{oh} + M_2{}' \cdot \begin{pmatrix} \log(k) \\ \log(k) \\ \log(k) \\ 0 \end{pmatrix}. \qquad \text{(A.5)}$$

If the subject remains still during video recording, k at each pixel is constant over time; therefore, the second term is constant, which means that the temporal variation is in the first term and the estimation error appears only in the constant term. In other words, the amplitude of temporal variation is not influenced by subject shape.
